# Association of Mobile Phone Location Data Indications of Travel and Stay-at-Home Mandates With COVID-19 Infection Rates in the US

**DOI:** 10.1001/jamanetworkopen.2020.20485

**Published:** 2020-09-08

**Authors:** Song Gao, Jinmeng Rao, Yuhao Kang, Yunlei Liang, Jake Kruse, Dorte Dopfer, Ajay K. Sethi, Juan Francisco Mandujano Reyes, Brian S. Yandell, Jonathan A. Patz

**Affiliations:** 1 GeoDS Lab, Department of Geography, University of Wisconsin–Madison, Madison; 2 School of Veterinary Medicine, University of Wisconsin–Madison, Madison; 3 School of Medicine and Public Health, University of Wisconsin–Madison, Madison; 4 Statistics and American Family Insurance Data Science Institute, University of Wisconsin–Madison, Madison

## Abstract

**Importance:**

A stay-at-home social distancing mandate is a key nonpharmacological measure to reduce the transmission rate of severe acute respiratory syndrome coronavirus-2 (SARS-CoV-2), but a high rate of adherence is needed.

**Objective:**

To examine the association between the rate of human mobility changes and the rate of confirmed cases of SARS-CoV-2 infection.

**Design, Setting, and Participants:**

This cross-sectional study used daily travel distance and home dwell time derived from millions of anonymous mobile phone location data from March 11 to April 10, 2020, provided by the Descartes Labs and SafeGraph to quantify the degree to which social distancing mandates were followed in the 50 US states and District of Columbia and the association of mobility changes with rates of coronavirus disease 2019 (COVID-19) cases.

**Exposure:**

State-level stay-at-home orders during the COVID-19 pandemic.

**Main Outcomes and Measures:**

The main outcome was the association of state-specific rates of COVID-19 confirmed cases with the change rates of median travel distance and median home dwell time of anonymous mobile phone users. The increase rates are measured by the exponent in curve fitting of the COVID-19 cumulative confirmed cases, while the mobility change (increase or decrease) rates were measured by the slope coefficient in curve fitting of median travel distance and median home dwell time for each state.

**Results:**

Data from more than 45 million anonymous mobile phone devices were analyzed. The correlation between the COVID-19 increase rate and travel distance decrease rate was –0.586 (95% CI, –0.742 to –0.370) and the correlation between COVID-19 increase rate and home dwell time increase rate was 0.526 (95% CI, 0.293 to 0.700). Increases in state-specific doubling time of total cases ranged from 1.0 to 6.9 days (median [interquartile range], 2.7 [2.3-3.3] days) before stay-at-home orders were enacted to 3.7 to 30.3 days (median [interquartile range], 6.0 [4.8-7.1] days) after stay-at-home social distancing orders were put in place, consistent with pandemic modeling results.

**Conclusions and Relevance:**

These findings suggest that stay-at-home social distancing mandates, when they were followed by measurable mobility changes, were associated with reduction in COVID-19 spread. These results come at a particularly critical period when US states are beginning to relax social distancing policies and reopen their economies. These findings support the efficacy of social distancing and could help inform future implementation of social distancing policies should they need to be reinstated during later periods of COVID-19 reemergence.

## Key Points

**Question:**

Did human mobility patterns change during stay-at-home orders and were the mobility changes associated with the coronavirus disease 2019 (COVID-19) curve?

**Findings:**

This cross-sectional study using anonymous location data from more than 45 million mobile phones found that median travel distance decreased and stay-at-home time increased across the nation, although there was geographic variation. State-specific empirical doubling time of total COVID-19 cases increased (ie, the spread reduced) significantly after stay-at-home orders were put in place.

**Meaning:**

These findings suggest that stay-at-home social distancing mandates were associated with the reduced spread of COVID-19 when they were followed.

## Teaser

This cross-sectional study uses anonymous mobile phone location data to examine travel and home dwell time patterns before and after enactment of stay-at-home orders in US states during the coronavirus disease 2019 (COVID-19) pandemic.

## Introduction

The coronavirus disease 2019 (COVID-19) pandemic is a global threat with escalating health, economic, and social challenges. As of April 11, 2020, there were 492 416 total confirmed cases and 18 559 total deaths in the US, according to reports from the Centers for Disease Control and Prevention (CDC).^1^ People are still witnessing widespread community transmission of COVID-19 all over the world. To date, there is neither a vaccine nor pharmacological agent found to reduce the transmission of severe acute respiratory syndrome coronavirus-2 (SARS-CoV-2), the virus that causes COVID-19. Thus, the effects of nonpharmacological pandemic control and intervention measures, including travel restrictions, closures of schools and nonessential business services, wearing of face masks, testing, isolation, and timely quarantine on delaying the spread of COVID-19, have been largely investigated and reported.^2,3,4,5,6^ To mitigate and ultimately contain the COVID-19 pandemic, one of the important nonpharmacological control measures to reduce the transmission rate of SARS-CoV-2 in the population is social (ie, physical) distancing. An interactive web-based mapping platform that provides timely quantitative information on how people in different counties and states reacted to state-at-home social distancing mandates has been developed (eAppendix 2 in the Supplement).^7^ It integrates geographic information systems and daily updated human mobility statistical patterns derived from millions of anonymized and aggregated smartphone location data at the county level in the US.^7,8,9,10^

Reduced mobility and trips may help limit people’s exposure to large in-person gatherings. However, it is worth noting that reduced mobility does not necessarily ensure that social distancing in practice follows the CDC’s definition: “stay at least 6 feet (about 2 arms’ length) from other people.”^11^ Due to the mobile phone Global Positioning System horizontal error and uncertainty,^12^ such physical distancing patterns cannot be directly identified from the user aggregated mobility data; that would require other wearable sensors or mobile phone Bluetooth trackers, which raise issues of personal data privacy and ethical concerns.^13^ Because COVID-19 is more contagious and far more deadly than seasonal flu,^14^ social distancing is critical in the fight to save lives and prevent illness. However, to what degree such guidelines have been followed from place to place before and after shelter-in-place orders across the US and the quantitative effect on flattening the curve are as yet unknown, to our knowledge.

To this end, we used 2 human mobility metrics, the median of individual maximum travel distance and stay-at-home time derived from location data from millions of mobile phones, to assess the association of stay-at-home policies with reducing the spread of COVID-19. For each state, we examined these measures against the rate of SARS-CoV-2 infection cases.

## Methods

A waiver of institutional review board review and informed consent was obtained from the University of Wisconsin–Madison because anonymized and aggregated data were used and our study does not involve human participants as defined. This study follows the Consolidated Health Economic Evaluation Reporting Standards (CHEERS) reporting guideline.

### Data

In this cross-sectional study, the epidemiological confirmed cases data were retrieved from the Corona Data Scraper open source project,^15^ which provides local-level and community-driven reports, and we conflated the data with the state-level department of health services official reports in each state to ensure the data quality. To understand how people reacted to the stay-at-home social distancing guidelines imposed during the COVID-19 pandemic, human mobility changes were considered in terms of changes in travel distance and stay-at-home dwell time. The travel distance mobility data were collected from an open-source repository released by Descartes Labs,^8^ while the home dwell time data derived from more than 45 million anonymous mobile phone users were processed from SafeGraph.^7^ Both data sources were acquired at the county level and aggregated to the state level using median and interquartile range (IQR) values. To consider the socioeconomic factors that may be associated with statewide changes in human mobility, socioeconomic variables at the state level were also collected from the American Community Survey^16^ and the US Census Bureau.^17^ The following socioeconomic variables and geospatial data sets were retrieved and computed: population (ie, number of people), population density (measured by population divided by area of state), proportion of population with bachelor’s degree, proportions of population of different races/ethnicities, proportion of population of different age groups, median household income, and urban core area boundaries.

### Statistical Analysis

Simple linear regression and multivariate linear regression analyses were performed using the scikit-learn package version 0.23.1 in Python. The Pearson correlation coefficient with 2-sided significance test, *P* < .05, was computed using the SciPy package version 1.4.0 in Python.

#### Curve Fitting for Pandemic Spread, Travel Distance, and Home Dwell Time

In the mathematical modeling process, we used a few types of mathematical formulas (eAppendix 1 in the Supplement) to fit the curve of the cumulative confirmed cases for the COVID-19 with respect to their temporal changes in each state and selected the following scaling law with a deivation term formula as the most appropriate: *y_c_*(*t*) = *t^b^* + *k,* in which *y_c_* is the total number of confirmed cases in each state as a function of time, *t* is the number of days from March 11, 2020 (when the COVID-19 became a pandemic), and *b* and *k* are parameters we will estimate. By fitting the curve, we can compare the infection rates among different states using the coefficient *b* estimated from the model. Meanwhile, we used linear regression to detect the travel distance decreasing rate (represented by the slope estimated from the linear model) over time (eFigure 2 in the Supplement) and examined whether there was a correlation between the increase rate of cases and the distance decreasing rate. We also fitted the curve for the home dwell time changes for each state using the linear regression model. The linear model was selected from a few different models because it is the simplest one, and results of all fitted models were similar. We then calculated the correlation between the home dwell time increasing rate (the slope estimated from the linear model) and the increase rate of the number of confirmed cases.

#### Evaluating Factors Associated With Changes in Travel Distance and Home Dwell Time

To understand what socioeconomic factors were associated with travel distance changes and home dwell time changes, a multilinear regression model integrating socioeconomic factors was used to fit the mobility change rates that were represented by the slope estimates for each state. The *R*^2^ as goodness of fit and significance of variables are reported (eAppendix 1 in the Supplement).

#### Calculating the Doubling Time of Total Confirmed Cases

We investigated how the social distancing guidelines and stay-at-home orders (eTable 5 in the Supplement) were associated with the pandemic doubling time of COVID-19 confirmed cases from March 11 to April 10, 2020, in each state. We used mathematical curve fitting models and mechanistic epidemic models (eAppendix 1 in the Supplement) using Bayesian parametric estimation of the serial interval distribution of successive cases to cross validate the conclusion.^18,19^ We calculated the doubling time of the number of cumulative confirmed cases (ie, the time intervals it takes for the cumulative confirmed cases to double in size^20^) to reflect the characteristics of the COVID-19 pandemic spread, especially how the stay-at-home orders in each state were associated with flattening the COVID-19 curve. The larger the doubling time, the smoother the pandemic increase curve. Within the time frame of our study, the state-level increase rates of COVID-19 cases in the US were either exponential or subexponential, thus we implemented an exponential model and a power-law model to fit the curve for calculating the doubling time. We also calculated the doubling time based on empirical observations (model-free) to further explore how the doubling time differs in these methods. We used the effective date of the stay-at-home order to split the confirmed case data into 2 parts: before the order and after the order. We fitted each model on the data before the order and after the order, then we calculated the doubling times of the confirmed cases based on the model and empirical COVID-19 infection data. The doubling time of the cumulative confirmed cases in each state is defined as
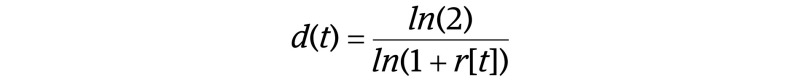
In which *d*(*t*) represents the doubling time of the cumulative confirmed cases on date *t* in each state, ln(x) is the natural log of *x*, and *r*(*t*) represents the increase rate of the cumulative confirmed cases on date *t* in each state.

In addition, we visualized and investigated the overall probability density distribution of the median doubling time before and after the stay-at-home order in each state to have a better understanding of the overall changes in the pandemic spread nationwide. Furthermore, we measured the similarity in probability density distribution of the median doubling time between the fitting results and the empirical data using the Jensen–Shannon Divergence.^21^

## Results

### Trends of Human Mobility Changes

**Figure 1.**
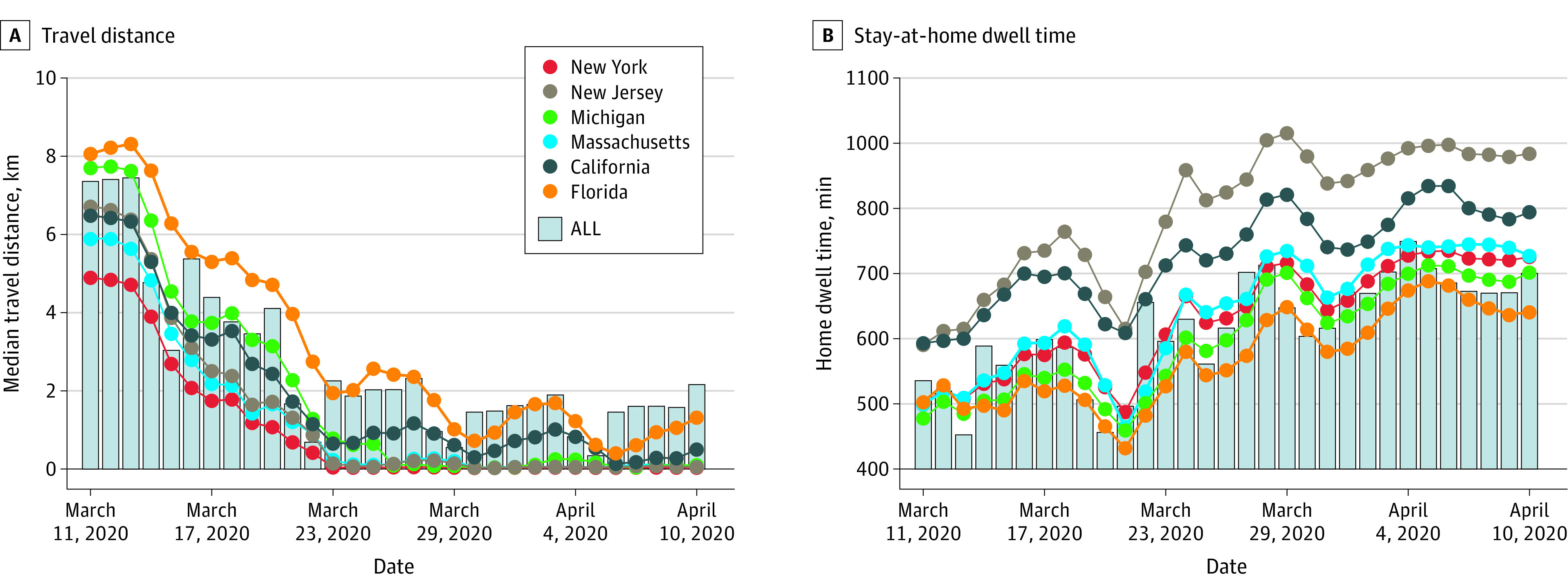
Temporal Changes in Median of Individual Maximum Travel Distance and Median Home Dwell Time in the Most Infected US States From March 11 to April 10, 2020

**Figure 2.**
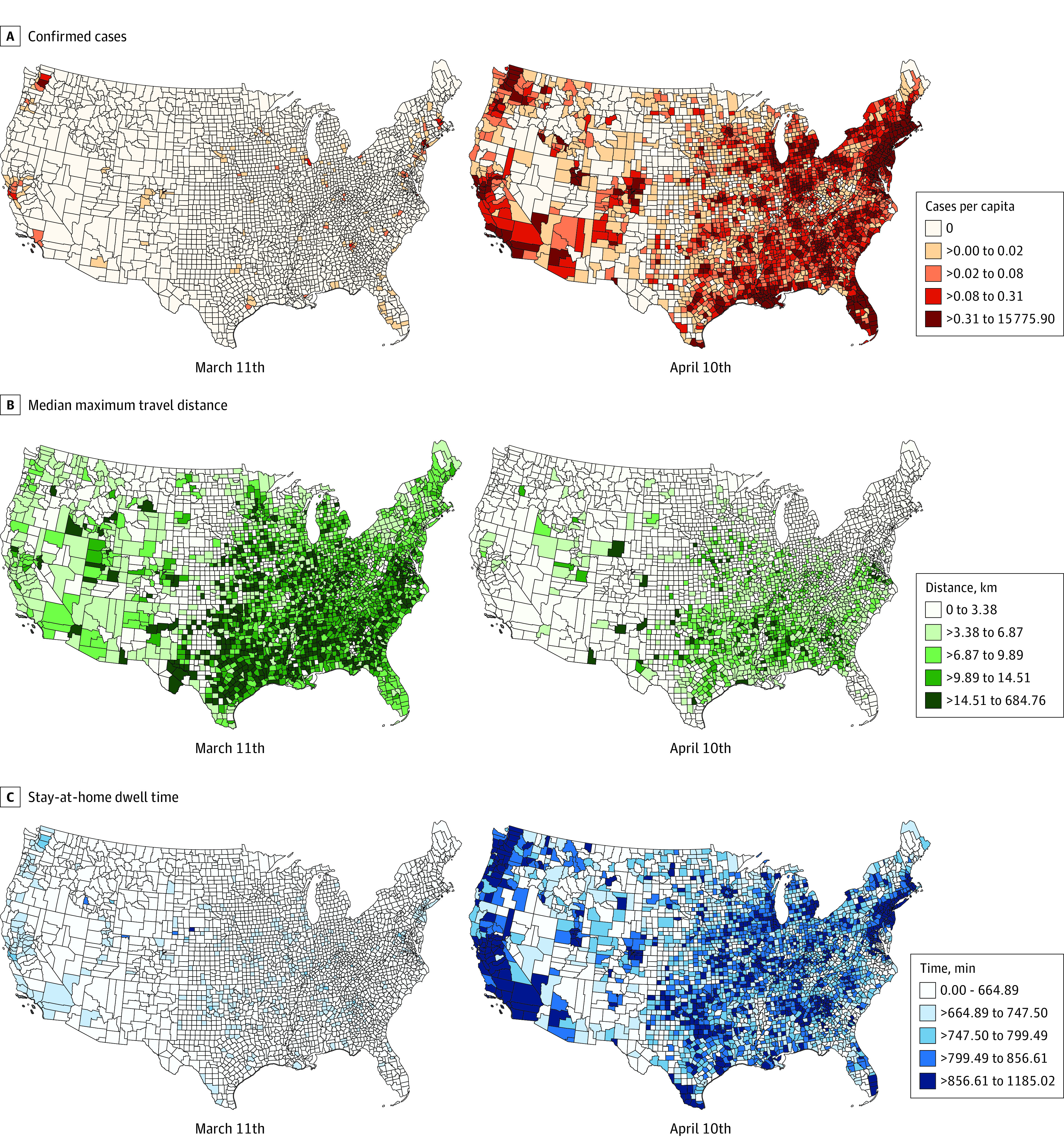
Comparison Among Confirmed Coronavirus Disease 2019 Cases Per Capita, Median of Individual Maximum Travel Distance, and Median Home Dwell Time From March 11 and April 10, 2020

Data from more than 45 million anonymous mobile phone devices were analyzed. The associations of stay-at-home policies with human mobility changes are illustrated in Figure 1, Figure 2, and the Table. Figure 1A shows the temporal changes of the median of individual maximum travel distances in the states with the highest infection rates (ie, New York, New Jersey, Michigan, California, and Massachusetts) by April 10, 2020. People’s daily mobility decreased significantly but with different temporal lags following the implementation of statewide stay-at-home orders across these states (Table). Figure 1B shows the state-specific temporal changes of median home dwell time. With the social distancing guidelines and shelter-at-home orders in place, the median home dwell time increased significantly in most states since March 23, 2020 (Table). Figure 2 shows the spatial distributions of confirmed cases per capita and the median of travel distances and median of home dwell time in 2 specific days as snapshots for comparison of mobility patterns with the COVID-19 infection rate before and after stay-at-home-orders: March 11 and April 10, 2020. The median travel distance decreased and the median home dwell time increased across the US during this period. In addition, we calculated the means of median travel distances before and after the stay-at-home-orders in each state. The median travel distances decreased in all states (Table). Implementation of stay-at-home social distancing policies were associated with human movement changes, that is, people generally reduced their daily travel distances and increased their home dwell time. Interestingly, the multiple linear regression model result for the increasing rate of home dwell time with the socioeconomic variables shows that the ratio of Asian individuals in each state was positively associated with longer home dwell time at the state level. The higher the proportion of Asian population, the longer the median home dwell time of residents in that state (eTable 7 in the Supplement).

**Table.** Empirical Doubling Time of Total Infected Cases and the Median Travel Distance and Home Dwell Time Before and After Stay-at-Home Orders

State	Doubling time, d			Travel distance, km			Home dwell time, min		
	Median (IQR)		Change	Median (IQR)		Change	Median (IQR)		Change
	Before order	After order		Before order	After order		Before order	After order	
Alabama	3.3 (2.3-4.4)	6.5 (5.4-7.8)	3.2	6.651 (5.356-8.278)	4.311 (4.283-4.903)	–2.328	660.9 (576.3-695.4)	781.4 (758.5-799.2)	120.5
Alaska	6.9 (3.5-9.2)	30.3 (23.6-38.9)	23.4	1.369 (0.168-2.450)	0.091 (0.049-0.092)	–1.273	342.3 (282.5-376.2)	427.1 (343.6-454.4)	84.8
Arizona	2.5 (2.0-3.8)	6.8 (5.8-10.5)	4.3	3.227 (1.82-5.071)	1.037 (0.878-1.274)	–2.231	523.9 (489.3-560.7)	637.9 (520.9-645.1)	114.0
California	3.3 (3.1-3.7)	5.3 (3.8-7.3)	2.0	3.922 (3.128-6.177)	0.770 (0.259-0.986)	–3.207	748.1 (642.7-760.4)	833.0 (754.4-874.0)	84.9
Colorado	2.6 (2.3-3.2)	6.2 (5.7-9.3)	3.6	2.824 (1.387-4.334)	0.319 (0.095-0.464)	–2.496	529.6 (475.1-548.0)	676.5 (575.7-692.6)	146.9
Connecticut	1.7 (1.3-2.8)	4.5 (2.8-7.5)	2.8	3.100 (2.174-4.482)	0.396 (0.107-0.549)	–2.687	667.5 (583.0-725.4)	822.5 (752.4-854.1)	155.0
Delaware	2.9 (1.5-4.7)	4.7 (3.7-5.4)	1.8	4.102 (2.888-5.704)	0.641 (0.183-0.937)	–3.462	629.3 (546.9-667.6)	749.5 (676.8-795.6)	120.2
Florida	3.0 (2.1-3.9)	10.0 (8.8-11.1)	7.0	3.484 (1.805-5.224)	0.930 (0.655-1.208)	–2.622	559.6 (476.2-604.4)	694.3 (680.0-717.5)	134.7
Georgia	3.5 (2.3-5.0)	6.4 (6.1-10.7)	2.9	4.852 (3.292-6.57)	2.278 (1.818-3.04)	–2.758	636.6 (546.2-674.1)	759.4 (732.3-784.3)	122.9
Hawaii	2.0 (1.6-2.4)	7.3 (5.2-11.1)	5.3	4.294 (3.131-6.057)	1.147 (1.054-1.466)	–3.177	625.7 (541.1-649.5)	789.4 (607.3-830.9)	163.7
Idaho	1.3 (1.0-2.6)	4.8 (2.9-9.8)	3.5	3.424 (2.661-4.599)	1.286 (1.063-1.713)	–2.208	567.9 (499.7-604.7)	686.2 (621.3-718.7)	118.3
Illinois	1.9 (1.9-2.4)	4.7 (4.0-7.1)	2.8	4.214 (3.046-6.604)	0.784 (0.427-1.144)	–3.428	648.6 (599.5-694.8)	764.0 (725.9-802.8)	115.4
Indiana	2.7 (2.0-3.0)	3.7 (3.0-4.2)	1.0	4.513 (3.41-6.232)	1.64 (1.432-2.193)	–2.932	605.2 (525.7-634.2)	718.8 (653.5-757.9)	113.6
Kansas	2.7 (1.8-3.5)	5.8 (5.1-10.2)	3.1	3.589 (2.300-4.657)	1.897 (1.735-2.269)	–1.67	606.2 (553.6-644.3)	702.1 (607.0-730.9)	96.0
Kentucky	2.5 (1.7-4.1)	5.4 (4.3-9.1)	2.9	4.778 (3.745-6.087)	2.802 (2.333-3.256)	–2.041	630.5 (562.8-670.4)	744.3 (686.1-764.2)	113.8
Louisiana	2.1 (1.9-2.3)	4.6 (3.1-8.7)	2.5	6.242 (5.925-8.289)	3.176 (2.877-3.830)	–3.122	609.5 (515.4-631.4)	736.9 (675.7-763.0)	127.4
Maine	3.7 (2.4-7.1)	16.5 (11.6-17.6)	12.8	2.413 (0.735-3.331)	0.361 (0.094-0.705)	–2.014	553.2 (481.6-590.2)	690.0 (638.8-703.4)	136.7
Maryland	2.8 (2.2-3.6)	4.2 (3.4-6.1)	1.4	2.346 (0.307-3.654)	0.122 (0.045-0.092)	–2.271	688.5 (611.4-745.6)	794.9 (676.6-824.0)	106.4
Massachusetts	3.8 (3.0-5.3)	4.7 (4.5-6.3)	0.9	2.323 (0.991-3.412)	0.108 (0.045-0.104)	–2.213	640.4 (538.5-670.0)	780.8 (692.0-812.3)	140.5
Michigan	2.3 (1.4-2.8)	4.4 (3.7-7.1)	2.1	3.562 (2.274-5.058)	0.104 (0.046-0.131)	–3.454	566.6 (492.3-600.5)	734.3 (649.8-764.3)	167.6
Minnesota	3.0 (1.7-4.9)	8.7 (7.6-9.4)	5.7	2.927 (1.359-4.268)	0.482 (0.138-0.509)	–2.54	556.9 (500.4-607.0)	701.4 (607.1-732.2)	144.5
Mississippi	2.8 (1.7-5.1)	9.4 (6.4-13.6)	6.6	7.103 (5.675-8.868)	4.751 (4.11-5.919)	–2.613	612.1 (514.0-654.5)	744.6 (720.4-767.7)	132.5
Montana	2.4 (1.8-3.2)	8.3 (7.4-14.5)	5.9	2.353 (1.821-2.953)	0.820 (0.405-1.158)	–1.475	443.3 (400.8-506.0)	559.6 (477.2-577.8)	116.3
Nevada	3.7 (1.7-5.0)	11.2 (8.5-12.6)	7.5	2.432 (0.687-4.353)	0.502 (0.253-0.764)	–1.962	516.0 (479.3-553.0)	611.5 (596.7-620.5)	95.6
New Hampshire	3.0 (2.3-4.3)	5.8 (4.3-11.7)	2.8	3.689 (1.527-5.603)	0.818 (0.266-1.073)	–3.014	585.0 (528.3-631.6)	735.4 (623.7-752.7)	150.4
New Jersey	1.8 (1.3-2.0)	4.2 (3.1-6.6)	2.4	3.244 (1.972-5.362)	0.095 (0.043-0.085)	–3.162	722.1 (671.7-819.5)	968.4 (900.8-983.9)	246.3
New Mexico	3.1 (2.6-3.5)	5.2 (4.4-6.9)	2.1	3.492 (2.728-4.579)	0.993 (0.873-1.275)	–2.519	467.8 (407.9-489.1)	577.5 (488.1-596.8)	109.8
New York	1.8 (1.5-2.2)	6.4 (4.4-9.5)	4.6	2.093 (1.137-3.554)	0.037 (0.032-0.039)	–2.056	580.0 (527.3-644.9)	767.4 (669.5-785.6)	187.4
North Carolina	2.7 (2.1-3.5)	6.3 (5.1-11.0)	3.6	5.220 (3.935-7.065)	2.679 (2.204-3.199)	–2.577	606.2 (545.8-633.2)	690.1 (595.4-711.2)	84.0
Ohio	2.1 (1.9-2.5)	5.3 (3.8-8.0)	3.2	4.076 (3.275-6.096)	1.202 (0.806-1.603)	–2.934	611.0 (547.3-653.0)	729.7 (688.0-762.5)	118.7
Oklahoma	2.4 (1.6-3.1)	5.6 (4.3-6.8)	3.2	5.962 (4.864-7.734)	3.550 (2.881-4.277)	–2.511	631.3 (563.2-664.9)	767.3 (707.3-804.4)	136.1
Oregon	3.8 (3.2-4.3)	6.7 (5.0-10.8)	2.9	2.667 (1.930-3.900)	0.571 (0.232-0.854)	–2.124	629.3 (575.8-663.4)	742.3 (687.0-789.9)	113.0
Pennsylvania	2.5 (2.1-3.3)	5.8 (4.5-6.0)	3.3	1.798 (0.078-2.445)	0.184 (0.089-0.246)	–1.609	656.0 (562.7-704.5)	776.7 (770.2-798.4)	120.6
Rhode Island	1.9 (1.4-3.5)	4.6 (4.1-5.3)	2.7	2.286 (0.804-3.592)	0.256 (0.071-0.357)	–2.034	705.1 (587.1-733.2)	795.2 (747.6-823.0)	90.1
South Carolina	2.4 (1.8-4.2)	5.8 (3.9-8.1)	3.4	6.484 (4.942-8.405)	3.898 (3.651-4.390)	–2.664	586.9 (532.3-619.1)	700.2 (626.4-712.3)	113.2
Tennessee	3.3 (1.8-4.0)	10.3 (8.4-12.5)	7.0	5.679 (4.094-7.368)	3.442 (3.215-4.098)	–2.189	647.8 (590.0-683.3)	731.4 (617.3-760.8)	83.6
Texas	3.4 (2.5-5.5)	6.0 (5.5-7.1)	2.6	4.076 (2.413-5.763)	1.869 (1.837-2.326)	–2.249	589.5 (525.1-645.8)	728.8 (722.2-759.4)	139.2
Utah	2.5 (1.9-4.1)	6.7 (5.2-11.6)	4.2	3.351 (2.094-4.933)	1.369 (0.916-1.791)	–2.05	642.6 (560.2-666.3)	710.9 (667.9-721.0)	68.3
Vermont	2.3 (1.6-2.8)	7.1 (5.1-11.9)	4.8	2.716 (0.843-4.386)	0.166 (0.059-0.201)	–2.592	465.8 (414.6-515.0)	648.4 (535.6-668.2)	182.7
Virginia	3.4 (2.4-4.9)	4.8 (4.1-7.2)	1.4	3.261 (1.454-4.669)	1.029 (0.627-1.320)	–2.273	607.6 (556.1-645.8)	695.1 (596.8-716.4)	87.5
Washington	4.5 (4.1-6.2)	12.3 (5.2-14.2)	7.8	2.710 (2.027-4.187)	0.253 (0.054-0.332)	–2.501	683.5 (618.6-717.0)	811.6 (760.0-848.3)	128.1
Washington, DC	3.5 (1.9-5.6)	6.9 (4.3-7.2)	3.4	0.85 (0.031-1.112)	0.026 (0.024-0.027)	–0.823	615.7 (523.4-639.7)	716.7 (696.9-722.0)	101.0
West Virginia	1.0 (1.0-1.3)	4.4 (3.9-7.3)	3.4	4.611 (3.573-6.217)	1.691 (1.345-2.095)	–2.939	586.3 (488.6-626.1)	693.1 (619.5-721.2)	106.8
Wisconsin	2.3 (1.9-2.6)	7.0 (6.1-9.6)	4.7	3.233 (2.061-4.871)	0.753 (0.574-1.23)	–2.477	594.2 (549.6-631.4)	720.2 (660.3-763.8)	126.0
Wyoming	3.1 (2.1-5.1)	7.9 (5.5-11.0)	4.8	2.719 (2.381-3.433)	1.798 (1.218-2.198)	–0.867	478.3 (430.9-539.2)	617.7 (491.8-636.6)	139.4

Abbreviations: DC, District of Columbia; IQR, interquartile range.

### Association of Rate of Infection With Mobility Changes

**Figure 3.**
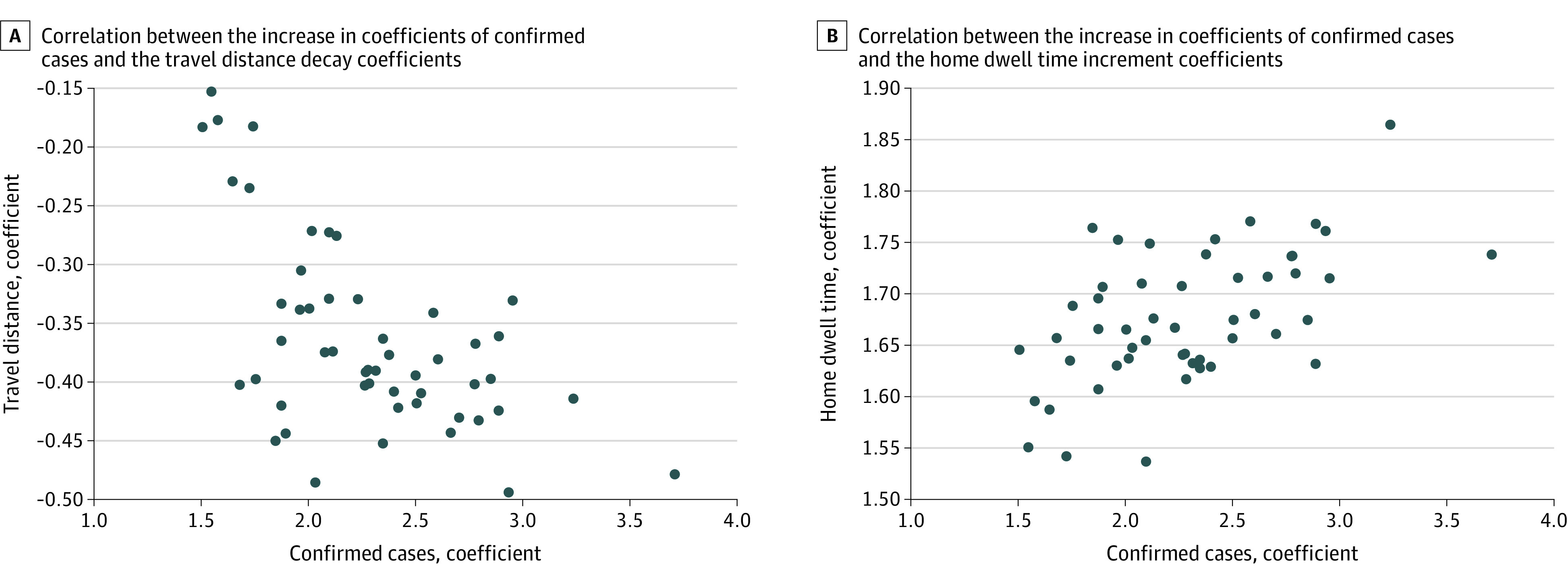
State-Level Correlation Between the Increase Coefficients of Confirmed Cases, Travel Distance Decay Coefficients, and Home Dwell Time Increase Coefficients

We fitted the curves for the state-specific COVID-19 confirmed cases using the scaling-law with a deviation term formula^22^ and identified the top 5 states with the largest increase rates of confirmed COVID-19 cases by April 10, 2020: New York, New Jersey, California, Michigan, and Massachusetts. Our fitting results corresponded to the up-to-date COVID-19 situation at that time (eTable 1 and eTable 2 in the Supplement). eFigure 1 in the Supplement shows the reported cases and the fitting curves in these 5 states using the scaling-law with a deviation term formula. The Pearson correlation coefficient between the cases increase rate and the distance decay rate was –0.586 (95% CI, –0.742 to –0.370; *P*  < .001) (eTable 3 in the Supplement). Figure 3A shows the state-level correlation between the increase coefficients of confirmed cases and the travel distance decay coefficients across the nation. The moderate negative correlation indicates that in the states where the confirmed cases were increasing faster, people generally reduced their daily travel distance more quickly.

Figure 3B shows the state-level correlation between the increase coefficients of confirmed cases and the home dwell time increment coefficients across the nation. The increase rates and the home dwell time rates (eTable 4 in the Supplement) had a positive correlation of 0.526 (95% CI, 0.293 to 0.700; *P* < .001), which suggests that in states with higher case increase rates, home dwell time of residents in this state were generally longer. These association analyses found that there was statistically significant mobility reduction associated with the increase rate of COVID-19 cases and that people in most states reduced their daily travel distance and increased stay-at-home time.

In addition, the statistical variation of the mobility measures can be largely explained (travel distance: *R*^2^ = 0.59; *P* < .001; home dwell time: *R*^2^ = 0.69; *P* < 001) by socioeconomic factors, including state policies, race/ethnicity, population density, age groups, and median household income (eAppendix 1, eTable 6, and eTable 7 in the Supplement). Recent studies have also identified partisan differences in individual responses to stay-at-home social distancing guidelines during the COVID-19 pandemic (H. Alcott et al, unpublished data, July 2020).

### Pandemic Doubling Time Changes

The fitted curves by an exponential model and a power-law model are shown in eFigure 3 and eFigure 4 in the Supplement. For the exponential model before the statewide stay-at-home orders, initial estimates of the increase rates of the number of confirmed cases for the pandemic in each state were 0.17 to 0.70 per day with a doubling time of 1.3 to 4.3 days (median [IQR], 2.6 [2.1-2.9] days). A similar result was found by fitting the power-law model, in which initial estimates of the case rates before the orders in each state were 0.12 to 0.71 cases per day with a doubling time of 1.3 to 6.2 days (median [IQR], 2.7 [2.2-3.1] days). The finding aligned well with the doubling time of 2.3 to 3.3 days in the early pandemic epicenter in Wuhan, China.^23^ After the implementation of stay-at-home orders, the estimates of the case rate in each state by the exponential model were reduced to 0.03 to 0.21 cases per day, with a doubling time increased to 3.7 to 27.7 days (median [IQR], 5.7 [4.7-6.9] days). Similarly, the estimates of the case rate in each state by the power-law model were reduced to 0.02 to 0.17 cases per day, with a doubling time increased to 4.3 to 29.8 days (median [IQR], 6.3 [5.4-7.9] days). The finding also aligned well (measured by Jensen–Shannon Divergence) with the result from the observed epidemiological data (Table), in which the empirical case rate in each state was 0.11 to 0.95 cases per day with a doubling time of 1.0 to 6.9 days (median [IQR], 2.7 [2.3-3.3] days) before the statewide stay-at-home orders, and reduced to 0.02 to 0.21 per cases day with a doubling time increased to 3.7 to 30.3 days (median [IQR], 6.0 [4.8-7.1] days) after the orders. The curve fitting results also matched the outcomes of mechanistic epidemic models (eFigure 7 in the Supplement), such as the models reported by Cori et al^18^ and Thompson et al.^19^ These models used confirmed cases and the serial interval, that is, the days between 2 successive infection cases.

**Figure 4.**
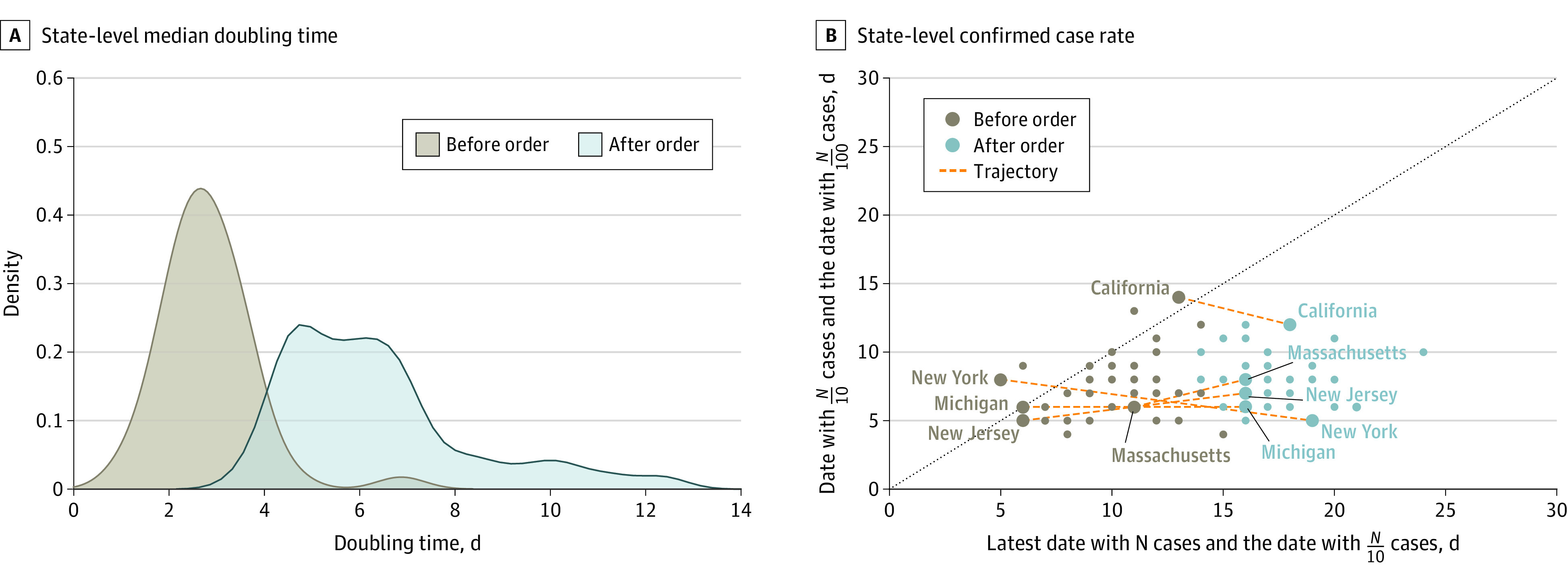
Probability Density Distributions and Ten-Hundred Plot of Coronavirus Disease 2019 Spread Before and After Stay-at-Home Orders B. The lower-right region represents subexponential growth; the diagonal line, exponential growth; and the upper left region, super-exponential growth. The top 5 states with the most confirmed cases are labeled and their change rate changes are visualized as trajectories. N indicates the number of coronavirus disease 2019 confirmed cases on that date.

In addition, we investigated the overall probability density distribution of the doubling time nationwide before and after the stay-at-home orders using the state-level median doubling time (Figure 4A; eFigure 5 and eFigure 6 in the Supplement). The doubling time nationwide increased after the stay-at-home orders (empirical observations: from median [IQR] 2.7 [2.3-3.3] days to median 6.0 [4.8-7.1] days). Our combined results on doubling times suggest that stay-at-home orders were associated with reduction of the COVID-19 pandemic spread and with flattening the curve. Similar findings have also been reported in a study by Sen et al^24^ on the association of stay-at-home orders with COVID-19 hospitalizations. In addition, the ten-hundred plot (Figure 4B)^25^ also shows that the case increase rate in each of the top 5 states (ie, New York, New Jersey, Michigan, California, and Massachusetts) slowed down after the stay-at-home orders (approaching subexponential growth). The statistical variation of the mobility measures can be largely explained (travel distance: *R*^2^ = 0.59; *P* < .05; home dwell time: *R*^2^ = 0.69; *P* < .05) (eAppendix 1 in the Supplement) by socioeconomic factors, including state policies, race/ethnicity, population density, age groups, and median household income (eTable 6 and eTable 7 in the Supplement). Recent studies have also identified partisan differences in individual responses to stay-at-home social distancing guidelines during the COVID-19 pandemic (H. Alcott et al, unpublished data, July 2020).

## Discussion

These findings suggest that stay-at-home social distancing mandates, when they were followed by measurable mobility changes, were associated with reduction in COVID-19 case rates. Great efforts have been made in scientific research communities on the study of human mobility patterns using various emerging data sources, including anonymized mobile phone call detail records,^26,27,28,29,30,31^ social media (eg, Twitter),^32,33^ location-based services, and mobile applications.^34,35,36,37,38^ During the COVID-19 pandemic, both individual-level and aggregated-level human mobility patterns have been found useful in pandemic modeling and digital contact tracing.^6,13,39,40^ However, technical challenges (eg, location uncertainty), socioeconomic and sampling bias,^41,42,43,44^ privacy and ethical concerns have been expressed by national and international societies.^45,46,47,48^ Moving forward, research efforts should continue exploring the balance of using such human mobility data at different geographic scales for public health and social good while preserving individual privacy and rights.

### Limitations

This study has some limitations. Potential confounding issues relate to other control measures, such as varying state-level quarantine protocols, availability of personal protective equipment, and timely testing, but the detailed information was not available, and the consistency of our results across most states makes such confounding less likely. In addition, the variability in the curve fitting estimated parameters was not accounted for the correlation analysis. There are variations in human behaviors and risk perception even within a state. All these factors contribute to the potential endogeneity of findings^49^ and the limitations.

## Conclusions

This cross-sectional study found a statistically significant association of 2 human mobility measures (ie, travel distance and stay-at-home time) with the rates of COVID-19 cases across US states. This study found a reduction of the spread of COVID-19 after stay-at-home social distancing mandates were enacted in most states. The findings come at a particularly critical period, when US states are beginning to reopen their economies but COVID-19 cases are surging. At such a time, our study suggests the efficacy of stay-at-home social distancing measures and could inform future public health policy making.

## Notes

Supplement. **eAppendix 1.** Supplementary Materials and Methods **eFigure 1.** Curve Fitting Results of Total Number of COVID-19 Cases for the Top 5 States With the Largest Coefficients **eFigure 2.** Linear Fit for Distance vs Days **eFigure 3.** Curve Fitting Results Using the Exponential Growth Model for Each State **eFigure 4.** Curve Fitting Results Using the Power-Law Growth Model for Each State **eFigure 5.** Overall Changes of the Doubling Time Nationwide Before and After Stay-at-Home Orders Using the Exponential Fitting Model **eFigure 6.** Overall Changes of the Doubling Time Nationwide Before and After the Stay-at-Home Orders Using the Power-Law Fitting Model **eFigure 7.** Empirical Observations of Confirmed Cases in the 45 States and the District of Columbia and the Projection of Cases Using the Mechanistic Prediction Model Before and After the Stay-at-Home Orders **eTable 1.** Coefficient and Mean Squared Error for Models Fitting the Confirmed Cases From March 11 to March 31 **eTable 2.** Top 10 States with Highest Coefficients for the Confirmed Cases Across 4 Models From March 11 to March 31 **eTable 3.** Top 5 States With the Largest Absolute Distance Coefficients From March 11 to March 31 **eTable 4.** Coefficient and Mean Squared Error for Models Fitting the Dwell Time at Home **eTable 5.** Order Type and Effective Date of Stay-At-Home Orders in Each State and the District of Columbia **eTable 6.** Regression Results of Travel Distance Changes at States **eTable 7.** Regression Results of Dwell Time at Home at States **eAppendix 2.** Mapping Videos **eReferences. **
