# Supplementary material for: Association of Mobile Phone Location Data Indications of Travel and Stay-at-Home Mandates With COVID-19 Infection Rates in the US
Source: JAMA Netw Open. 2020 Sep 8;3(9):e2020485. doi: 10.1001/jamanetworkopen.2020.20485 (PMC7489834; doi:10.1001/jamanetworkopen.2020.20485)
Supplement: Supplement. — eAppendix 1. Supplementary Materials and Methods eFigure 1. Curve Fitting Results of Total Number of COVID-19 Cases for the Top 5 States With the Largest Coefficients eFigure 2. Linear Fit for Distance vs Days eFigure 3. Curve Fitting Results Using the Exponential Growth Model for Each State eFigure 4. Curve Fitting Results Using the Power-Law Growth Model for Each State eFigure 5. Overall Changes of the Doubling Time Nationwide Before and After Stay-at-Home Orders Using the Exponential Fitting Model eFigure 6. Overall Changes of the Doubling Time Nationwide Before and After the Stay-at-Home Orders Using the Power-Law Fitting Model eFigure 7. Empirical Observations of Confirmed Cases in the 45 States and the District of Columbia and the Projection of Cases Using the Mechanistic Prediction Model Before and After the Stay-at-Home Orders eTable 1. Coefficient and Mean Squared Error for Models Fitting the Confirmed Cases From March 11 to March 31 eTable 2. Top 10 States with Highest Coefficients for the Confirmed Cases Across 4 Models From March 11 to March 31 eTable 3. Top 5 States With the Largest Absolute Distance Coefficients From March 11 to March 31 eTable 4. Coefficient and Mean Squared Error for Models Fitting the Dwell Time at Home eTable 5. Order Type and Effective Date of Stay-At-Home Orders in Each State and the District of Columbia eTable 6. Regression Results of Travel Distance Changes at States eTable 7. Regression Results of Dwell Time at Home at States eAppendix 2. Mapping Videos eReferences. [file jamanetwopen-e2020485-s001.pdf]

Supplementary Online Content

Gao S, Rao J, Kang Y, et al. Association of mobile phone location data indications of travel and stay-at-home mandates with COVID-19 infection rates in the US. *JAMA Netw Open*. 2020;3(9):e2020485. doi:10.1001/jamanetworkopen.2020.20485

eAppendix 1. Supplementary Materials and Methods
eFigure 1. Curve Fitting Results of Total Number of COVID-19 Cases for the Top 5 States With the Largest Coefficients
eFigure 2. Linear Fit for Distance vs Days
eFigure 3. Curve Fitting Results Using the Exponential Growth Model for Each State
eFigure 4. Curve Fitting Results Using the Power-Law Growth Model for Each State
eFigure 5. Overall Changes of the Doubling Time Nationwide Before and After Stay-at-Home Orders Using the Exponential Fitting Model
eFigure 6. Overall Changes of the Doubling Time Nationwide Before and After the Stay-at-Home Orders Using the Power-Law Fitting Model
eFigure 7. Empirical Observations of Confirmed Cases in the 45 States and the District of Columbia and the Projection of Cases Using the Mechanistic Prediction Model Before and After the Stay-at-Home Orders
eTable 1. Coefficient and Mean Squared Error for Models Fitting the Confirmed Cases From March 11 to March 31
eTable 2. Top 10 States with Highest Coefficients for the Confirmed Cases Across 4 Models From March 11 to March 31
eTable 3. Top 5 States With the Largest Absolute Distance Coefficients From March 11 to March 31
eTable 4. Coefficient and Mean Squared Error for Models Fitting the Dwell Time at Home
eTable 5. Order Type and Effective Date of Stay-At-Home Orders in Each State and the District of Columbia
eTable 6. Regression Results of Travel Distance Changes at States
eTable 7. Regression Results of Dwell Time at Home at States
eAppendix 2. Mapping Videos
eReferences

This supplementary material has been provided by the authors to give readers additional information about their work.

© 2020 Gao S et al. *JAMA Network Open.*

eAppendix 1. Supplementary Materials and Methods

Travel Distance Mobility Data and Home Dwell Time Data

The travel distance mobility data are collected from an open-source repository released by the Descartes Labs [10], while the home dwell time data are collected from SafeGraph [1]. Both datasets are collected through tracking usages of mobile phone apps of sampled users. By aggregating large-scale (over 45 million) anonymized location data from smartphones, the average individual movement pattern for a given area can be derived. To enhance privacy, individual data are de-identified and aggregated and all analyses are performed at aggregated spatial units (e.g., census block groups, counties, and states).

Travel distance mobility changes are derived using an index which measures the median of the maximum travel distances for all individuals in a given county on a given day [10, 3]. Such data are widely used to represent the dramatic human mobility changes in reaction to the COVID-19 [6, 3, 11].

To measure home dwell time, the home place for each individual is identified and the minutes for all sampled devices staying at that home place across the day are summed up. Median home dwell time for all observed devices is then aggregated in geographical units.

Fitting the epidemic growth curves

As the confirmed cases of COVID-19 keep growing, we aim to find a model that can describe such growth rates with respect to their temporal changes in different states. Therefore, we use four types of formulas to fit the curve of the confirmed cases for the COVID-19 epidemic. By fitting the curve, we can compare the growth rates among different states using the coefficients estimated from the model. The four models we tested are:

$$y_c(t) = t^b + k \tag{1}$$

$$y_c(t) = t^b \tag{2}$$

$$y_c(t) = at^b + k \tag{3}$$

$$y_c(t) = ae^{bt} \tag{4}$$

Where y_c is the total number of confirmed cases in each state as a function of time, t is the number of days from March 11 where the novel Coronavirus disease became a pandemic, a,b,k are parameters we will estimate.

We fit the curve for two time periods: March 11 to March 31 and March 11 to April 10 with consideration of the starting date variation of stay-at-home orders in each state (in Table S5) and the incubation period ranging from 1-14 days with a median of 4 days according to clinical characteristics of COVID-19 patients [5], as well as the testing capacity at the beginning and the variability of exponential or sub-exponential growth of the COVID-19 cases [8]. We analyzed two periods respectively. The fitting results for the period March 11 to March 31 fit better due to the fact that the majority changes of human responses in terms of mobility and home dwell time were observed after March 23, 2020 and with a short time lag across different states.

The results of fitting the curve using the four formulas from March 11 to March 31 are listed in Table S1. The coefficient b is used to reflect the growth rate of the confirmed cases. We use the Mean Square Error (MSE) as the goodness of fit measure for the curve fitting. Based on the MSE, the third formula $y_c(t) = at^b + k$ has the best fit curve for total number of confirmed cases in each state. The first formula and the fourth one have close goodness of fit and the second formula has the worst performance. We then looked at the coefficients for each state and Table S2 shows the top 10 states with the largest coefficients from each formula. A larger coefficient reflects a faster growth rate, so the top states in the Table S2 should be those that experienced the most rapid outbreaks of the COVID-19. When examining the top most infected states for the four models, the results for $y_c(t) = t^b + k$ and $y_c(t) = t^b$ better match the empirical observations in the study period. The top states from the two models that have the fastest growth rate are New York, New Jersey, California, and Michigan. Therefore, we use the coefficients generated from the approximate sub-exponential growth function $y_c(t) = t^b + k$ as the indices representing the epidemic growth rate. Figure S1 shows the cases and the fitted curves using formulas $y_c(t) = t^b + k$.

Fitting the mobility change rates

In addition to fitting the epidemic growth curve, we also fit the curve for travel distance and home dwell time changes. More specifically, we calculate the coefficient that describes the overall trend of the median of max-distance individual mobility at each state since March 11, 2020. The coefficient found by fitting the distance curve is used to measure the speed of changes to daily mobility patterns. The analysis is conducted for each state and we fit the distance decay pattern over days from March 11 using a linear regression model:

$$y_d(t) = bt + k \quad (5)$$

Where y_d is travel distance as a function of time, and t is the number of days from March 11, 2020. The coefficient of the travel distance change is represented using the slope parameter b. In addition, we processed the travel distance data to cut off the days where the distances are smaller than 0.1 km for 3 consecutive days. The reason to do so is that the plot of distance vs. days has a long tail for some areas because most people stayed at home in late March in response to COVID-19, known as the social distancing inertia [4]. Since we try to capture the most dramatic change of potential travel distance decrease in response to the COVID-19, we don't include the data that have distances ≤ 0.1 km for three consecutive days. An example of fitting the distance data with and without removing the distance ≤ 0.1 km is shown in Figure S2. For some states that responded quickly and reduced the travel distance, we can capture the quick decreasing travel distance in this way. Table S3 shows the top five states with the largest absolute distance decreasing rates. From this analysis, Michigan, Washington D.C., and New York, respectively, are the states that responded quickest to COVID-19 in terms of decreased mobility.

Furthermore, after we estimate the growth rate of confirmed cases and the decreasing rate of travel distance changes, we can discover whether there exists some correlation between them. The Pearson's correlation coefficient between the two coefficients using the data from March 11 to March 31 is -0.586 with a p-value <0.001, and the 95% confidence interval (CI) is -0.742 to -0.370. Figure 3A shows the two coefficients for all 50 states and DC. This negative relationship indicates that people have responded quickly to increases in confirmed cases. In places where COVID-19 cases are growing faster, people usually are quicker to reduce mobility and stay at home.

We also describe the relationship between home dwell time (in minutes) and the number of confirmed cases by estimating the coefficients that represent the change of the home dwell time and compute the correlation between the dwell time coefficient and the cases coefficient. The following three types of formulas are used to fit the curve of home dwell time (y) vs. the days from March 11 (t).

$$y(t) = t^b + k \quad (6)$$

$$y(t) = ae^{bt} \quad (7)$$

$$y(t) = bt + k \quad (8)$$

Table S4 shows the results of fitting home dwell time. The coefficients represent human mobility changes from the perspective of how long people stay at home. The three formulas all have positive coefficients, meaning that as time goes by, home dwell time is increasing. The three formulas all have close MSE and we choose the linear fit $y(t) = bt + k$ to represent the rate of change in home dwell time as we prefer a simpler formula when all formulas perform similarly.

We then compute the correlation between the home dwell time coefficients and the confirmed cases coefficients to detect whether such mobility changes are associated with the growth of COVID-19 cases. Figure 3B shows that the growth rates of cases in each state and the associated dwell time fitted coefficients have a positive correlation of 0.526 (95% CI: [0.293, 0.700], p-value < 0.001).

In addition, the correlation between the cases growth coefficients and the distance decay coefficients from the period March 11 to April 10 is very similar to the result of that for the period March 11 to March 31: -0.582 (95% CI: [-0.740, -0.365], p-value <0.001). Because we processed the distance data to cut off the days where the distance is < 0.1km and we can capture the distance decay rate even when the study period extended. For the correlation between the the cases growth coefficients and the home dwell time coefficients, however, the

correlation is 0.322 (95% CI: [0.051, -0.549], p-value <0.05), it is much smaller than the correlation we calculated for the period March 11 to March 31.

Evaluating factors influencing changes in travel distance and home dwell time

To understand how socio-economic factors influence changes in travel distance and home dwell time, we employed a multi-linear regression model involving the above-mentioned socio-economic variables as independent variables to model the mobility changes. The previously regressed change rates of travel distance b_d (Equation 5) and home dwell time b_t (Equation 8) are taken as the dependent variable of the following model:

$$b_{(d,t)} = a_0 + a_1x_1 + a_2x_2 + a_3x_3 + ... + a_nx_n \quad (9)$$

where $x_1,x_2,...,x_n$ are socio-economic factors that may influence these behaviour changes; and $a_0,a_1,...,a_n$ are coefficients which illustrate to what degree these independent variables contribute to the behaviour changes. Since spatial scale plays an important role in socio-economic metrics, we aggregated and analyzed data at the state level. Although we perform the linear regression in two time periods, from March 11 to March 31, and from March 11 to April 10, only the results of former one are reported. Because there were more substantial mobility changes (both travel distance and home dwell time) during the former time period. It is worth noting that, at the state level, records with median travel distance less than 0.1 km are removed. In addition, in terms of different age groups and race groups, the proportion of population over age 65 and the proportion of other race groups are assigned as the reference group in the experiments.

Table S6 shows the regression results for travel distance changes at the state level. The goodness-of-fit of the regression models is evaluated using R-squared (R^2) and p-value. The R-squared is 0.59 for the regression, and the model is statistically significant (p-value < 0.05). Results show that behaviour changes can be largely explained by state policies and socio-economic variables. Further investigation into other confounding factors associated with travel distance reduction is necessary to quantify the effects of those covariates.

Table S7 illustrates the regression results for home dwell time changes in response to socio-economic factors. The R-squared for multi-linear regression is 0.66 at state-level and the p-value is less than 0.05, which indicates that the results are statistically significant. In particular, the ratio of Asians has a positive standardized coefficient (6.959), p-value < 0.05, while the ratio of Hawaiians has a negative standardized coefficient (-2.863), p-value < 0.05, and both are significant at the state level. Accordingly, it can be inferred that the ratio of Asians is strongly correlated with longer home dwell time at the state level. The higher the proportion of Asian population, the longer the median home dwell time of residents in that state.

Calculating the epidemic growth doubling time

Based on the relationship between the number of confirmed cases and the date, we calculate the doubling time of the number of total confirmed cases (i.e., the time it takes for the confirmed cases to double in size) to reflect the characteristics of the COVID-19 epidemic growth. Additionally, we want to explore how the social distancing policy (e.g., stay-at-home orders) in each state makes a difference in flattening the curve. That is, the larger the doubling time, the less steep the epidemic growth curve. At the time of writing, the growth rates of COVID-19 cases in the U.S. states are either exponential or sub-exponential. We implement both of them to fit the curve for calculating the doubling time. We also calculated the doubling time based on empirical observations (model-free) to further explore how the doubling time differs in these methods. The two models we used are the exponential model (Equation 11) and the power-law model (Equation 12):

$$y_c(t) = I(0)e^{at} \quad (10)$$

$$y_c(t) = bt^a + I(0) \quad (11)$$

Where y_c is the total number of confirmed cases in each state; t represents the number of the days after the first case was confirmed in the state; $I(0)$ represents the number of confirmed cases on the first-case day; a,b are the

© 2020 Gao S et al. *JAMA Network Open.*

coefficients of the models. Note that we introduced the intercept term $I(0)$ into the two models to ensure that the initial case number on the first day is accurate. This is important since the accuracy of the thereafter calculated daily growth rate and doubling time relies on the number of initial cases.

We investigate how stay-at-home orders in each state associated with the doubling time of confirmed cases in that state. Within the time frame of our study, as shown in Table S5, there were five states (North Dakota, South Dakota, Nebraska, Iowa, and Arkansas) which didn't issued a stay-at-home order, and three states (Oklahoma, Utah, and Wyoming) which only had partial orders issued locally by cities or counties. All other states, as well as the District of Columbia, issued the order for all residents. South Carolina issued partial orders effective March 26, and then issued the full order effective April 7. We focus on the confirmed case data from March 11 to April 10, and we use the effective date of the stay-at-home order to split the confirmed case data into two parts: before the order and after the order. For the states with only partial orders, we use the earliest order effective date in that state. For South Carolina, we also use the earliest effective date (i.e., March 26). We investigate the doubling time in the states with statewide orders (except for Missouri since the effective date of the stay-at-home order in this state is the latest, and we don't have enough after-order data in this state given the date range of our study) and the District of Columbia. We fit the models on the data before the order and after the order respectively, and then we calculate the doubling time of the confirmed cases based on the two models.

The doubling time of the confirmed cases is defined as:

$$d(t) = \frac{ln(2)}{ln(1 + r(t))} \tag{12}$$

Where $d(t)$ stands for the doubling time of the cumulative infection cases on date t in each state; $r(t)$ represents the growth rate of the cumulative infection cases on date t in each state, and is defined as:

$$r(t) = \frac{I(t) - I(t-1)}{I(t-1)} \tag{13}$$

Where $I(t)$ represents the number of cumulative infection cases on date t. For the exponential model, the daily growth rate is constant, and thus the doubling time is constant and can be calculated as:

$$d_e = \frac{ln(2)}{a} \tag{14}$$

Where d_e is the doubling time of the exponential model, and a is the exponent parameter in the exponential model (Equation 10). Note that Equation (14) can be regarded as a special case of Equation (12). For the power-law model (Equation 11), since the doubling time is not constant, we first manually calculated the time-dependent growth rate of the predicted cases by the model in each day for each state. Specifically, for each date except for the starting date, we subtract the previous date's predicted case number from the current date's predicted case number, then divided by the previous date's predicted case number, thereby obtaining the time-dependent daily growth rate $r(t)$ (Equation 13). We then use the time-dependent growth rate to calculate the corresponding time-dependent doubling time of the power-law model, and we use the median of the time-dependent doubling time to represent the doubling time in that state during the before or after order period.

For the two curve fitting models, the fitting results are shown in Figures S3 and S4. The green dashed line in each subplot represents the fitted curve on the data before the stay-at-home order in the state; the blue curve represents the fitted curve on the data after the stay-at-home order in the state; the vertical black dashed line indicates the date when the stay-at-home order takes effect in each state. All the states showed a large initial growth rate and small doubling time in the number of total confirmed cases. However, the growth starts to slow down in general after the effective date of the stay-at-home order in each state, which shows the efficiency of the social distancing policy in suppressing the transmission of the novel coronavirus.

For empirical observations, we manually calculated the time-dependent growth rate of the reported cumulative confirmed cases in each day for each state. The empirical growth rate is also calculated following the same process as the power-law model. For each date except for the starting date, we subtract the previous date's total confirmed case number from the current date's total confirmed case number, then divided by the

previous date's total confirmed case number, thereby obtaining the time-dependent daily empirical growth rate. We then use the empirical time-dependent growth rate to calculate the corresponding empirical time-dependent doubling time, and we use the median of the time-dependent doubling time to represent the empirical doubling time in that state.

In addition, we visualize and investigate the overall probability density distribution of the median doubling time before and after the order in each state to have a better understanding of the overall changes in the epidemic growth nationwide. We applied Kernel Density Estimation (KDE) on doubling times in all the states to derive the overall probability density distribution, where the doubling time for each state is calculated by the median of the daily growth rate in that state. Note that the doubling time for the exponential model is a constant, so the median equals that constant. As is shown in Figure 4A, S5 and S6, the doubling time of confirmed cases nationwide has significantly increased after the order in each state (empirical observations: from median 2.7 days, IQR: 1.0 to median 6.0 days, IQR: 2.3; exponential model: from median 2.6 days, IQR: 0.8 to median 5.7 days, IQR: 2.2; power-law model: from median 2.7 days, IQR: 0.9 to median 6.3 days, IQR: 2.5)), which reinforce the point that stay-at-home orders, or social distancing policy, associated with the reduced spread of the virus when they are compliant. Furthermore, we measure the difference in probability density distribution between the fitting results and the real data using Jensen–Shannon Divergence (JSD). The JSD value ranges from 0 to 1 for two probability distributions; small JSD values indicate high similarity between two probability distributions. Compared with the probability density distribution of real data shown in Figure 4A, both the fitting results by the exponential model and power-law model show high similarity (i.e., with small JSD values) to the empirical data before the order (the exponential model: 0.11; the power-law model: 0.07) and after the order (the exponential model: 0.17; the power-law model: 0.18), respectively. This means that, despite different models, the discoveries are consistent: stay-at-home orders did associate with the slowdown of the COVID-19 case growth and with the increase of doubling time.

Mechanistic prediction models

The exponential equation approach was particularly suitable during the early outbreak phase but the sub-exponential growth fitted better after the city lock-downs [8], stay-at-home orders and following the social distancing regulations. Our curve fitting results matched the outcomes of mechanistic prediction models (Figure S7), such as the models reported by [2, 9]. The daily number of cumulative confirmed cases per U.S. state and the serial interval (SI), that is the number of days between two consecutive cases, were used assuming an average SI of 7.5 days with standard deviations (SD): 3.4 days as reported by [7], who estimated the SI using Bayesian parametric estimation from data in the city of Wuhan in China. In addition, the SI average and SD were sampled from normal distributions of mean 7.5 and SD 0.2, as well as SD mean of 3.4 and SD of 0.4 to sample from more SI distributions at random. Estimates of the instantaneous basic reproduction number (R0) were simulated by moving an 8-day sliding time interval across all data points since the start of the outbreak per state respectively. The instantaneous R0 on the last day before the start of the stay-at-home orders and from April 10, 2020, that is after the order went in, were used for projections of the confirmed number of cases per day. The resulting graphs are shown in Figure S7 for all states respectively where black dots represent observed values and the red dots represent the projected values for daily total confirmed cases. It shows that projected daily total confirmed cases from before the start date of the stay-at-home social distancing orders increase much more rapidly compared to the projected number of total confirmed cases starting April 10, 2020, that is after the orders went in. In summary, the mathematical curve fitting models and the mechanistic epidemic prediction models draw the same conclusion.

© 2020 Gao S et al. *JAMA Network Open.*

eFigure 1. Curve Fitting Results of Total Number of COVID-19 Cases for the Top 5 States With the Largest Coefficients

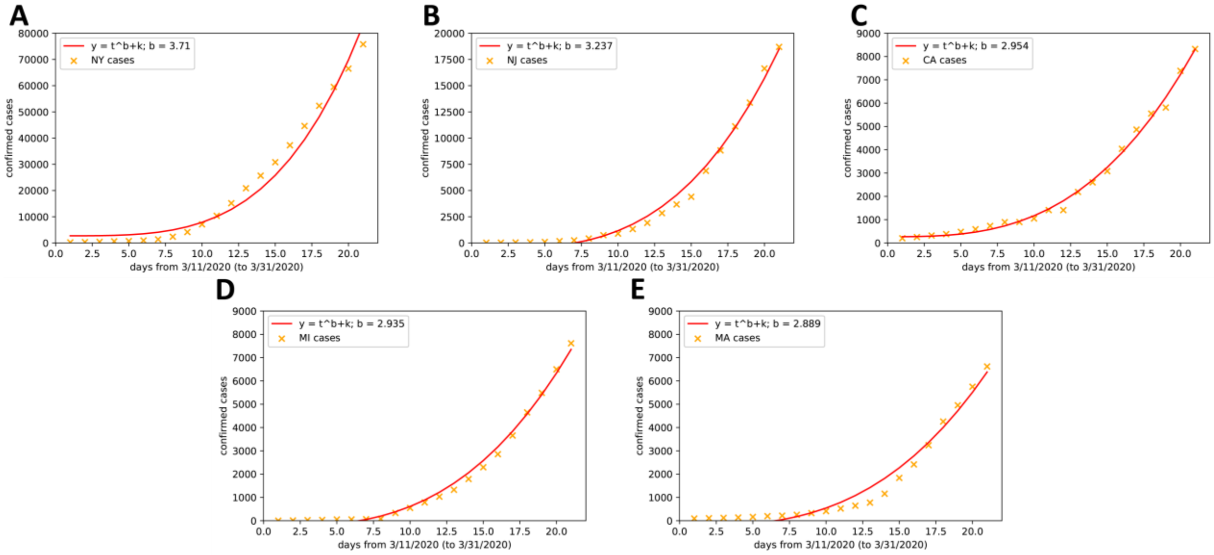

A: New York; B: New Jersey; C: California; D: Michigan; E: Massachusetts.

© 2020 Gao S et al. *JAMA Network Open.*

eFigure 2. Linear Fit for Distance vs Days

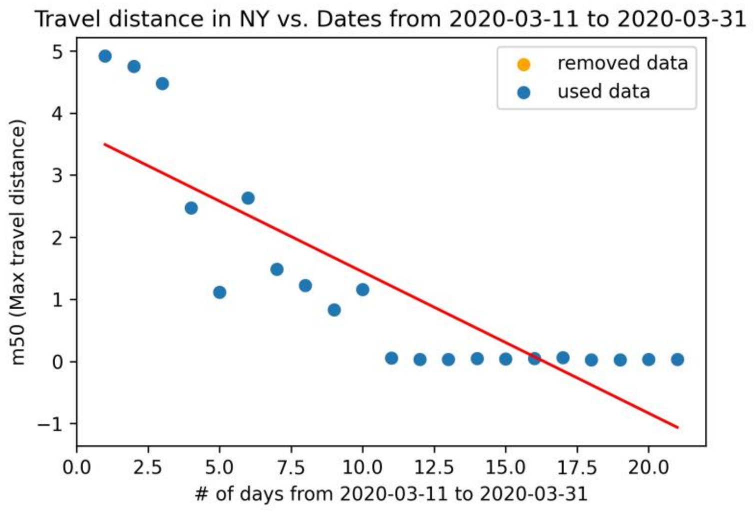

(a)

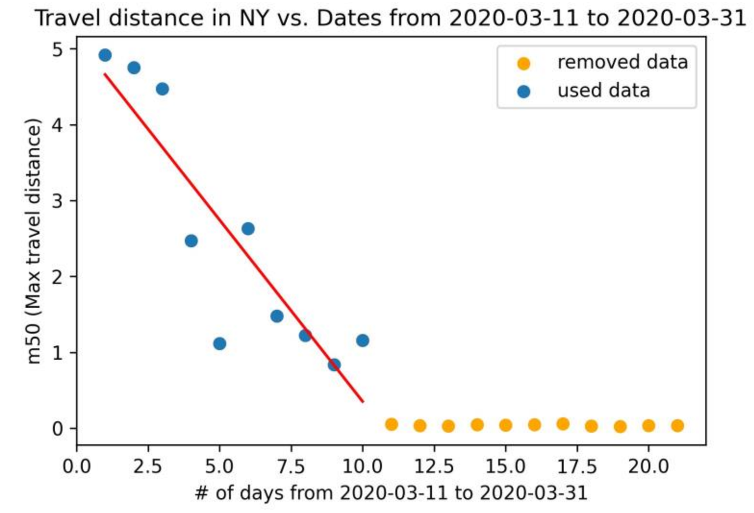

(b)

(a) using the original data and (b) using the data removing distance ≤ 0.1 km in New York state from March 11 to March 31.

© 2020 Gao S et al. *JAMA Network Open.*

eFigure 3. Curve Fitting Results Using the Exponential Growth Model for Each State

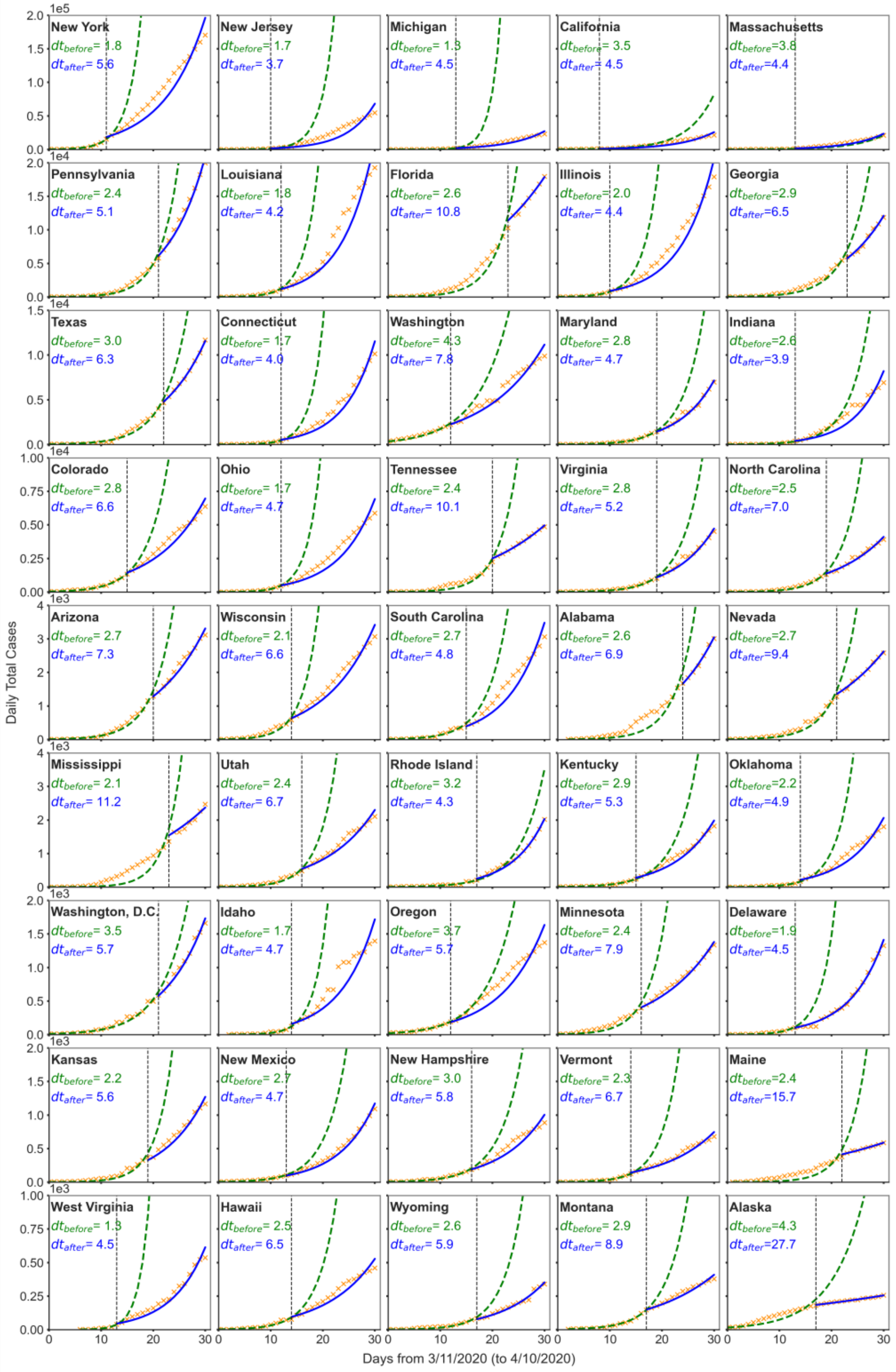

The green dashed line and the blue line represent the fitted curves on the data before and after the stay-at-home orders in each state, respectively; the vertical black dashed line indicates the effective date of the stay-at-home orders in each state. dt_{before} and dt_{after} represent the median doubling time before and after the order in each state.

© 2020 Gao S et al. *JAMA Network Open.*

eFigure 4. Curve Fitting Results Using the Power-Law Growth Model for Each State

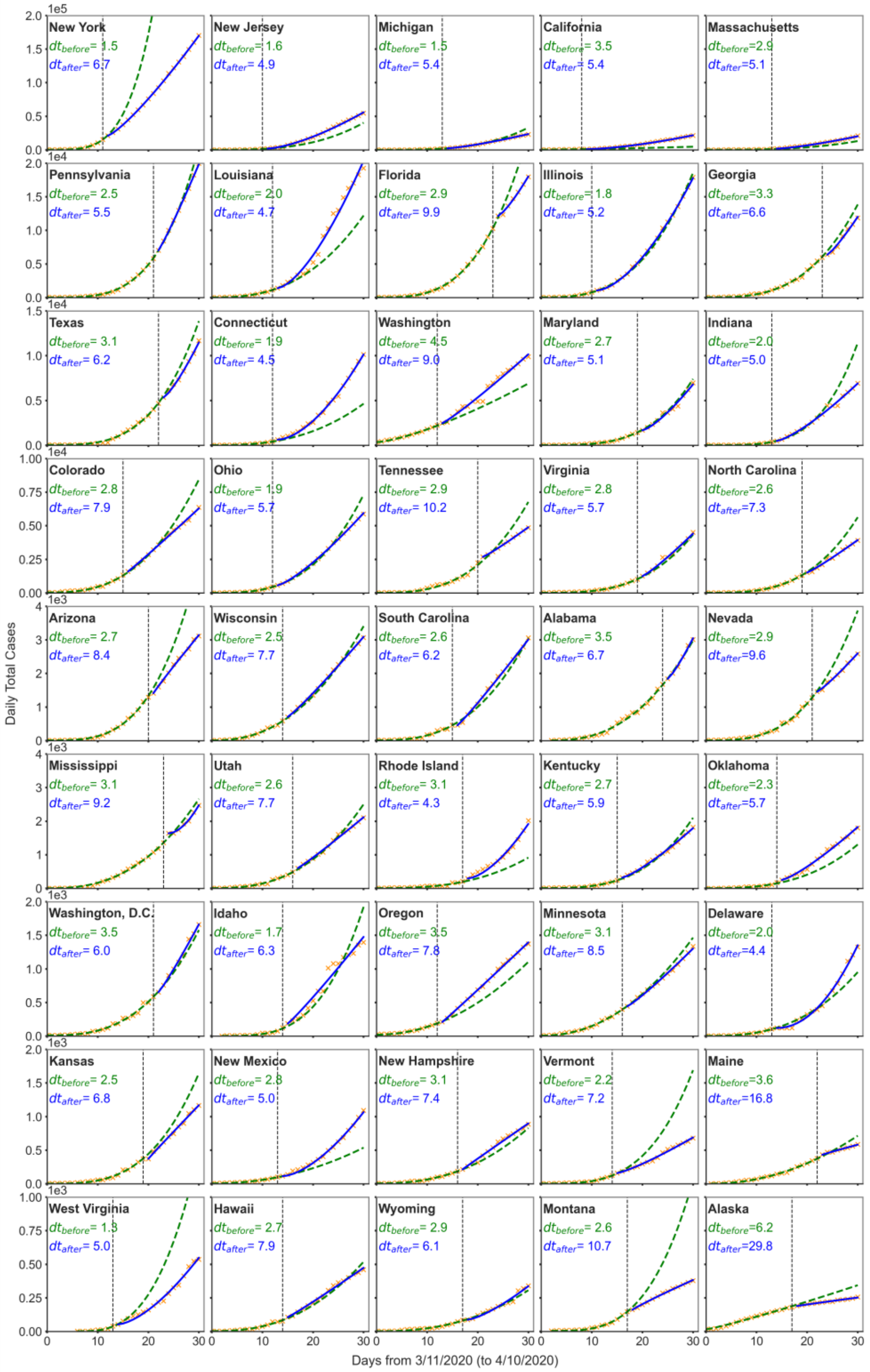

The green dashed line and the blue line represent the fitted curves on the data before and after the stay-at-home order in each state, respectively; the vertical black dashed line indicates the effective date of the stay-at-home order in each state. dt_{before} and dt_{after} represent the median doubling time before and after the order in each state.

© 2020 Gao S et al. *JAMA Network Open.*

eFigure 5. Overall Changes of the Doubling Time Nationwide Before and After Stay-at-Home Orders Using the Exponential Fitting Model

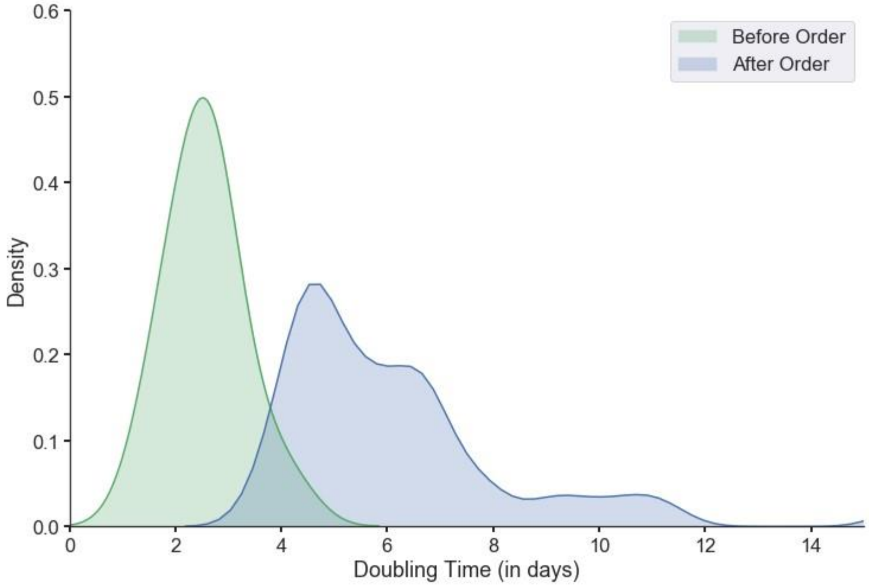

Figure S5: The overall changes of the doubling time nationwide before and after the order using the exponential fitting model.

© 2020 Gao S et al. *JAMA Network Open.*

eFigure 6. Overall Changes of the Doubling Time Nationwide Before and After the Stay-at-Home Orders Using the Power-Law Fitting Model

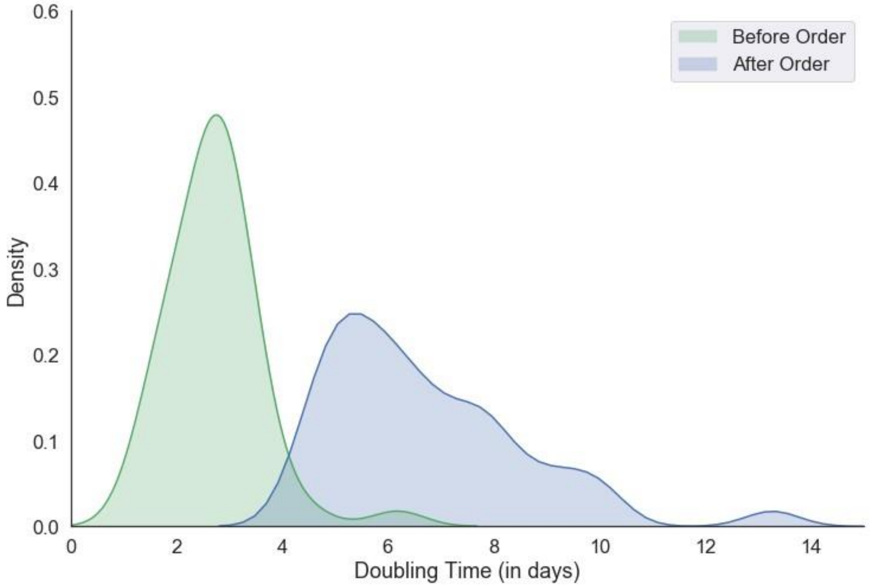

© 2020 Gao S et al. *JAMA Network Open.*

eFigure 7. Empirical Observations of Confirmed Cases in the 45 States and the District of Columbia and the Projection of Cases Using the Mechanistic Prediction Model Before and After the Stay-at-Home Orders

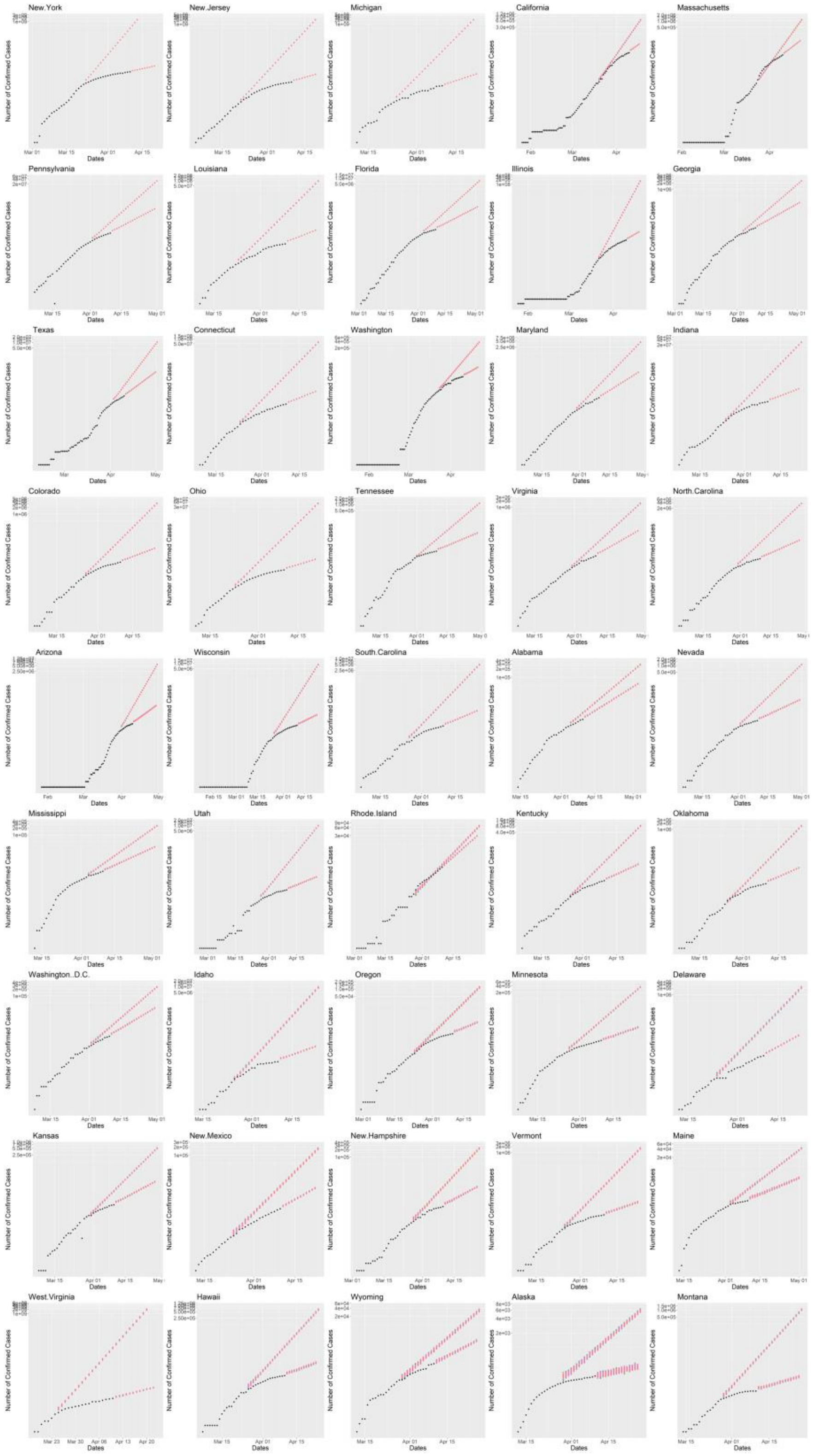

© 2020 Gao S et al. *JAMA Network Open.*

eTable 1. Coefficient and Mean Squared Error for Models Fitting the Confirmed Cases From March 11 to March 31

The fitting method	Coefficient b (mean)	MSE
$y_c = t^b + k$	2.284	2.694E+05
$y_c = t^b$	2.259	3.779E+05
$y_c = at^b + k$	3.210	4.336E+04
$y_c = ae^{bt}$	0.191	2.960E+05

© 2020 Gao S et al. *JAMA Network Open.*

eTable 2. Top 10 States with Highest Coefficients for the Confirmed Cases Across 4 Models From March 11 to March 31

$y_c = t^b + k$	$y_c = t^b$	$y_c = at^b + k$	$y_c = ae^{bt}$
New York	New York	Rhode Island	Rhode Island
New Jersey	New Jersey	Idaho	Idaho
California	California	Indiana	Indiana
Michigan	Michigan	Pennsylvania	Pennsylvania
Massachusetts	Florida	Massachusetts	New Jersey
Florida	Massachusetts	New Jersey	Missouri
Illinois	Washington	Maryland	Massachusetts
Louisiana	Illinois	Iowa	Texas
Washington	Louisiana	Missouri	Maryland
Pennsylvania	Pennsylvania	Texas	Michigan

© 2020 Gao S et al. *JAMA Network Open.*

eTable 3. Top 5 States With the Largest Absolute Distance Coefficients From March 11 to March 31

State	Distance coefficient
Michigan	-0.494
Washington, D.C.	-0.485
New York	-0.478
Missouri	-0.452
Delaware	-0.450

© 2020 Gao S et al. *JAMA Network Open.*

eTable 4. Coefficient and Mean Squared Error for Models Fitting the Dwell Time at Home

The fitting method	Coefficient b	MSE
$y = t^b + k$	1.677	4850.671
$y = ae^{bt}$	0.767	4791.662
$y = bt + k$	8.608	4864.841

© 2020 Gao S et al. *JAMA Network Open.*

eTable 5. Order Type and Effective Date of Stay-At-Home Orders in Each State and the District of Columbia

State Name	Order Type	Earliest Effective Date
California	Full	3/19/2020
Illinois	Full	3/21/2020
New Jersey	Full	3/21/2020
New York	Full	3/22/2020
Connecticut	Full	3/23/2020
Louisiana	Full	3/23/2020
Ohio	Full	3/23/2020
Oregon	Full	3/23/2020
Washington	Full	3/23/2020
Delaware	Full	3/24/2020
Indiana	Full	3/24/2020
Massachusetts	Full	3/24/2020
Michigan	Full	3/24/2020
New Mexico	Full	3/24/2020
West Virginia	Full	3/24/2020
Hawaii	Full	3/25/2020
Idaho	Full	3/25/2020
Oklahoma	Partial	3/25/2020
Vermont	Full	3/25/2020
Wisconsin	Full	3/25/2020
Colorado	Full	3/26/2020
Kentucky	Full	3/26/2020
South Carolina*	Partial, Full	3/26/2020, 4/7/2020
Minnesota	Full	3/27/2020
New Hampshire	Full	3/27/2020
Utah	Partial	3/27/2020
Alaska	Full	3/28/2020
Montana	Full	3/28/2020
Rhode Island	Full	3/28/2020
Wyoming	Partial	3/28/2020
Kansas	Full	3/30/2020
Maryland	Full	3/30/2020
North Carolina	Full	3/30/2020
Virginia	Full	3/30/2020
Arizona	Full	3/31/2020
Tennessee	Full	3/31/2020
Washington, D.C.	Full	4/1/2020
Nevada	Full	4/1/2020
Pennsylvania	Full	4/1/2020
Maine	Full	4/2/2020
Texas	Full	4/2/2020
Florida	Full	4/3/2020
Georgia	Full	4/3/2020
Mississippi	Full	4/3/2020
Alabama	Full	4/4/2020
Missouri	Full	4/6/2020
North Dakota	No	-
South Dakota	No	-
Nebraska	No	-
Iowa	No	-

© 2020 Gao S et al. *JAMA Network Open.*

Arkansas	No	-

“Full” means the order is issued for all residents. “Partial” means the order is issued locally by cities or counties. “No” means there is currently no stay-at-home order in this state.
*South Carolina issued partial orders effective March 26, and then issued the full order effective April 7. We use the earliest effective date for South Carolina.

© 2020 Gao S et al. *JAMA Network Open.*

eTable 6. Regression Results of Travel Distance Changes at States

Variables	Coefficients	Standard Error	z–value	p–value	Standardized Coefficients
Intercept	1.116	2.513	0.444	0.660	-0.364
Population	0.000	0.000	-1.688	0.100	-0.022
Median Age	-0.025	0.029	-0.853	0.399	-0.053
Population Density	0.000	0.000	-1.177	0.247	-0.026
Household Income	0.000	0.000	1.042	0.304	0.014
Proportion of Population under Age 18	-2.438	2.801	-0.870	0.390	-0.051
Proportion of Population between Age 18 to 44	-1.680	2.133	-0.788	0.436	-0.041
Proportion of Population between Age 45 to 64	-3.147	1.670	-1.884	0.067	-0.057
Proportion of Population over Age 65	0.000				0.000
Proportion of Whites	1.029	0.623	1.652	0.107	0.140
Proportion of Blacks	0.676	0.583	1.160	0.253	0.073
Proportion of Asians	2.786	1.399	1.991	0.054	0.153
Proportion of Natives	2.473	0.954	2.593	0.014	0.066
Proportion of Hawaiian	-3.191	2.250	-1.419	0.164	-0.046
Proportion of Other Race Groups	0.000				0.000
Proportion of Population with Bachelor's Degree	1.024	0.961	1.065	0.294	0.023
R^2: 0.59, p-value < 0.05					

© 2020 Gao S et al. *JAMA Network Open.*

eTable 7. Regression Results of Dwell Time at Home at States

Variables	Coefficients	Standard Error	z–value	p–value	Standardized Coefficients
Intercept	61.218	70.426	0.869	0.390	8.814
Population	0.000	0.000	-1.476	0.148	-0.544
Median Age	-0.716	0.824	-0.868	0.391	-1.508
Population Density	0.003	0.002	1.327	0.193	0.825
Household Income	-0.007	0.008	-0.907	0.370	-0.351
Proportion of Population under Age 18	-39.540	78.514	-0.504	0.618	-0.825
Proportion of Population between Age 18 to 44	-79.376	59.786	-1.328	0.192	-1.925
Proportion of Population between Age 45 to 64	-7.773	46.809	-0.166	0.869	-0.142
Proportion of Population over Age 65	0.000				0.000
Proportion of Whites	26.084	17.457	1.494	0.144	3.551
Proportion of Blacks	25.046	16.326	1.534	0.134	2.715
Proportion of Asians	126.581	39.213	3.228	0.003	6.959
Proportion of Natives	25.069	26.732	0.938	0.354	0.672
Proportion of Hawaiian	-200.726	63.055	-3.183	0.003	-2.863
Proportion of Other Race Groups	0.000				0.000
Proportion of Population with Bachelor's Degree	-20.269	26.941	-0.752	0.457	-0.449

R^2: 0.66, p-value < 0.05

© 2020 Gao S et al. *JAMA Network Open.*

eAppendix 2. Mapping Videos

(1) Mapping the COVID-19 infected areas in the U.S., available at:

"https://geods.geography.wisc.edu/wp-content/uploads/2020/04/US_cases_animation_March.mov" (2) Mapping human mobility changes at the U.S. county level, available at:

"https://geods.geography.wisc.edu/wp-content/uploads/2020/04/US_mobilitychanges_animation. mp4"

© 2020 Gao S et al. *JAMA Network Open.*

eReferences

[1] SafeGraph Inc. Accessed April 12, 2020, https://www.safegraph.com/.

[2] A. Cori, N. M. Ferguson, C. Fraser, and S. Cauchemez. A new framework and software to estimate timevarying reproduction numbers during epidemics. *American Journal of Epidemiology*, 178(9):1505–1512, 2013.

[3] S. Gao, J. Rao, Y. Kang, Y. Liang, and J. Kruse. Mapping county-level mobility pattern changes in the united states in response to covid-19. *SIGSPATIAL Special*, 12(1):16–26, 2020.

[4] S. Ghader, J. Zhao, M. Lee, W. Zhou, G. Zhao, and L. Zhang. Observed mobility behavior data reveal social distancing inertia. *arXiv preprint arXiv:2004.14748*, 2020.

[5] W.-j. Guan, Z.-y. Ni, Y. Hu, W.-h. Liang, C.-q. Ou, J.-x. He, L. Liu, H. Shan, C.-l. Lei, D. S. Hui, et al. Clinical characteristics of coronavirus disease 2019 in China. *New England Journal of Medicine*, 2020.

[6] J. R. Hipp and A. Boessen. The shape of mobility: Measuring the distance decay function of household mobility. *The Professional Geographer*, 69(1):32–44, 2017.

[7] R. Li, S. Pei, B. Chen, Y. Song, T. Zhang, W. Yang, and J. Shaman. Substantial undocumented infection facilitates the rapid dissemination of novel coronavirus (SARS-CoV2). *Science*, 2020.

[8] B. F. Maier and D. Brockmann. Effective containment explains sub-exponential growth in confirmed cases of recent COVID-19 outbreak in Mainland China. *Science*, 2020.

[9] R. Thompson, J. Stockwin, R. van Gaalen, J. Polonsky, Z. Kamvar, P. Demarsh, E. Dahlqwist, S. Li, E. Miguel, T. Jombart, et al. Improved inference of time-varying reproduction numbers during infectious disease outbreaks. *Epidemics*, 29:100356, 2019.

[10] M. S. Warren and S. W. Skillman. Mobility changes in response to covid-19. *arXiv preprint arXiv:2003.14228*, 2020.

[11] L. Zhang, S. Ghader, M. L. Pack, C. Xiong, A. Darzi, M. Yang, Q. Sun, A. Kabiri, and S. Hu. An interactive covid-19 mobility impact and social distancing analysis platform. *medRxiv*, 2020.

© 2020 Gao S et al. *JAMA Network Open.*
